# Settable Polymeric Autograft Extenders in a Rabbit Radius Model of Bone Formation

**DOI:** 10.3390/ma14143960

**Published:** 2021-07-15

**Authors:** Lauren A. Boller, Madison A.P. McGough, Stefanie M. Shiels, Craig L. Duvall, Joseph C. Wenke, Scott A. Guelcher

**Affiliations:** 1 Department of Biomedical Engineering, Vanderbilt University, 2201 West End Ave, Nashville, TN 37235, USA; lauen.a.boller@vanderbilt.edu (L.A.B.); madison.a.mcgough@gmail.com (M.A.P.M.); craig.duvall@vanderbilt.edu (C.L.D.); 2 U.S. Army Institute of Surgical Research, 3698 Chambers Rd, San Antonio, TX 78234, USA; stefanie.m.shiels.ctr@mail.mil (S.M.S.); joseph.c.wenke.civ@mail.mil (J.C.W.); 3 Department of Chemical and Biomolecular Engineering, Vanderbilt University, 2201 West End Ave, Nashville, TN 37235, USA; 4 Vanderbilt Center for Bone Biology, Vanderbilt University Medical Center, 1211 Medical Center Dr., Nashville, TN 37212, USA

**Keywords:** autograft extender, bone, polyurethane

## Abstract

Autograft (AG) is the gold standard for bone grafts, but limited quantities and patient morbidity are associated with its use. AG extenders have been proposed to minimize the volume of AG while maintaining the osteoinductive properties of the implant. In this study, poly(ester urethane) (PEUR) and poly(thioketal urethane) (PTKUR) AG extenders were implanted in a 20-mm rabbit radius defect model to evaluate new bone formation and graft remodeling. Outcomes including µCT and histomorphometry were measured at 12 weeks and compared to an AG (no polymer) control. AG control examples exhibited new bone formation, but inconsistent healing was observed. The implanted AG control was resorbed by 12 weeks, while AG extenders maintained implanted AG throughout the study. Bone growth from the defect interfaces was observed in both AG extenders, but residual polymer inhibited cellular infiltration and subsequent bone formation within the center of the implant. PEUR-AG extenders degraded more rapidly than PTKUR-AG extenders. These observations demonstrated that AG extenders supported new bone formation and that polymer composition did not have an effect on overall bone formation. Furthermore, the results indicated that early cellular infiltration is necessary for harnessing the osteoinductive capabilities of AG.

## 1. Introduction

Autograft (AG) bone is considered the gold standard in bone grafting. It is osteoinductive, osteoconductive, and osteogenic, and it does not pose a risk for disease transmission [1,2,3]. AG comes in various forms including both cancellous and cortical [3]. Cancellous AG is most often harvested from the iliac crest (IC); however, other donor sites such as the posterior superior iliac spine, femur, proximal tibia, and distal radius are utilized [4,5,6,7]. Cancellous AG contains mesenchymal stem cells (MSCs), osteoblasts, and growth factors including bone morphogenetic proteins (BMPs), which contribute to its osteoinductivity [3,8]. The trabeculae present within cancellous AG allow for enhanced cellular infiltration and vascularization in comparison to cortical AG [8]. Cortical AG is ideal for defects that require structural support as it offers superior mechanical properties compared with cancellous AG. However, cortical AG is less osteoinductive than cancellous AG, and its density results in slower revascularization and inhibits cellular infiltration [8,9]. Despite its osteogenic properties, AG is a scarce resource with multiple drawbacks including donor site morbidity (10–39% of patients), limited availability, the need for a second surgical site [6], and rapid resorption dependent on the bone density and embryologic origin of the AG [10].

The use of allograft from donors is an alternative to AG. Allograft is more readily available than AG and provides structural support, but it does not possess the same osteoinductive capacity as AG due to its processing [2]. Furthermore, allograft faces potential immune rejection and slow osseointegration with host bone [11]. Synthetic materials such as recombinant human bone morphogenic proteins (rhBMPs) have emerged as substitutes for AG [12,13,14], but none of these alternatives has been shown to match all of the benefits provided by AG. Furthermore, the use of the FDA-approved rhBMP-2 treatment (INFUSE^®^ bone graft, Medtronic) is limited to a few clinical indications [15,16,17].

To overcome the limitations in AG including availability and rapid resorption, various approaches to increase the overall volume of AG while maintaining its osteogenic and osteoinductive properties have been employed. Clinically, AG is typically blended with an ‘extender’ to reduce the volume of AG needed for implantation [18,19]. An early study demonstrated the utility of demineralized bone matrix as an AG extender [20] More recently, tissue engineered approaches to incorporate synthetic bone substitutes with AG have been investigated. Calcium phosphates (CaPs) such as β-tricalcium phosphate (β-TCP) and hydroxyapatite were evaluated as AG extenders for spinal applications [21,22,23,24]. Similarly, poly(propylene fumarate)- and poly(lactide-co-glycolide) (PLGA)-based polymer AG extenders have also been evaluated for spinal applications [25,26,27,28], while AG extenders utilizing bioactive glass particles have been investigated in the femur [29].

Lysine-based poly(ester urethanes) (PEURs) and poly(thioketal urethanes) (PTKURs) have been previously investigated in bone regeneration applications [30,31,32,33]. The mechanical properties of these materials can be easily altered, and the addition of ceramic particles, AG, and allograft supports new bone formation at various anatomic sites [34,35,36,37]. Previous work has demonstrated selective, cell-mediated, first-order degradation of PTKUR in vivo [38]. Furthermore, low-porosity PTKURs utilized in rabbit intertransverse processes [39] and femoral plugs [33] exhibited new bone formation, but minimal PTKUR degradation was observed. Slow degradation is advantageous in applications in which mechanical stability is required; however, in applications utilizing biologics, faster graft resorption is necessary to harness the osteoinductivity. In a previous study, PEUR was used to deliver rhBMP-2 and demonstrated balanced polymer resorption and new bone formation [34,40]. Therefore, we compared PEUR [41,42] with PTKUR [38] as an AG extender to test the hypothesis that faster degrading PEUR would support increased cellular infiltration and bone formation in a rabbit radius model.

Herein, settable and resorbable PTKUR-AG and PEUR-AG extenders were implanted into a 20 mm critical-sized segmental defect in the rabbit radius to investigate the effects of polymer composition on cellular infiltration, new bone formation, and polymer resorption. In this study, PTKUR or PEUR was blended with fresh IC AG and the resulting material subsequently molded to size and implanted in the defect. In vivo outcomes assessed post-operatively with X-ray, µCT, histology, and histomorphometry were compared to an AG control.

## 2. Materials and Methods

### 2.1. Materials

All chemicals were purchased from Sigma-Aldrich (St. Louis, MO, USA) with the exception of anhydrous diethyl ether purchased from Fisher Scientific. Lysine triisocyanate-polyethylene glycol (LTI-PEG) prepolymer (NCO = 21.7%) was obtained from Ricerca Biosciences LLC (Concord, OH, USA).

### 2.2. Polyester Triol and Thioketal Diol Synthesis

The polyester triol (molecular weight 450 g mol^−1^) was synthesized utilizing a previously published method [43]. Briefly, glycerol, 70% ε-caprolactone, 20% glycolide, and 10% DL-lactide monomers were mixed for 40 h under argon at 140 °C. The resulting fluid was vacuum dried at 80 °C for 48 h. Thioketal (TK) diol was synthesized utilizing a previously published method [32]. Briefly, 2,2-dimethoxypropane and thioglycolic acid were reacted in the presence of bismuth (III) chloride at room temperature for 24 h. The resulting solution was filtered, dissolved in tetrahydrofuran, and added dropwise to LiAlH_4_ under anhydrous conditions. The reaction was refluxed at 52 °C for 18 h and the product filtered and vacuum dried for 48 h.

### 2.3. AG Extender Fabrication

PTKUR- and PEUR-AG extenders were fabricated by adapted two-component reactive-liquid molding methods as previously described [39]. Briefly, polyisocyanate comprised of either TK diol or polyester triol, 10 pphp iron acetylacetonate (FeAA) catalyst in ϵ-caprolactone 0.5% (*w*/*w*), and LTI-PEG prepolymer were mixed together. Morselized AG (70 wt%) was added to the mixture and stirred by hand until homogeneous. The resulting mixture was injected as a viscous paste that was cured to form a solid implant in situ. The targeted index (NCO:OH) was 200.

### 2.4. AG Extenders in a Rabbit Radius Defect

**Figure 1:**
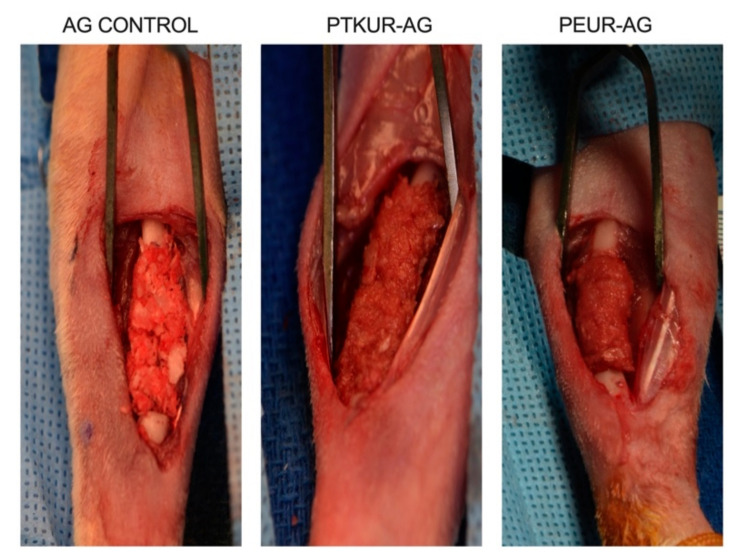
Surgical images. AG control, PTKUR-AG extender, and PEUR-AG extender in the 20 mm defect prior to closure.

Adult New Zealand White rabbits were used in this study (*n* = 12). The protocol was approved by the Ethics Committee of the U.S. Army Institute of Surgical Research (A-18-035). Animals were randomly assigned to PEUR-AG, PTKUR-AG, or AG control treatment groups (*n* = 4 per group). Assuming an effect size of 0.999 (determined from a previous study [41]) and alpha of 0.05, an a priori power analysis determined that a sample size of *n* = 3 would provide a power of 0.95. Thus, 4 animals per group were considered to provide sufficient power for this study. Animals were premedicated with slow-release buprenorphine (0.1 mg kg^−1^) and anesthetized with isoflurane (1–3%). For all groups, the animal’s left hindlimb and right forelimb were shaved and prepared for sterile surgery using alternating washes of alcohol and povidone-iodine. The left IC was exposed, and AG (0.6–0.7 g) was harvested using an oscillating saw. Excess soft tissue was removed and a bone mill (R. Quétin) was used to morselize the harvested bone. The IC harvest site was closed and the right radius exposed. An oscillating saw was used to create a 20 mm segmental defect in the radius. AG extenders were prepared as explained above and shaped to size (5 mm × 20 mm). AG control (morselized AG without PTKUR or PEUR) was molded to shape and carefully placed within the defect. A surgical elevator was used to place the AG extenders in the defects to ensure correct placement. AG extenders were allowed to cure in situ (Figure 1) after which the radial site was closed. Post-operative X-ray images (Faxitron X20) were taken throughout recovery and Calcein green and Xylenol orange fluorochromes were injected at 4 and 8 weeks post-operatively, respectively, to evaluate bone remodeling temporally. Animals were anesthetized and euthanized at 12 weeks. The radii were harvested and placed into formalin for further analysis.

### 2.5. µCT Analysis

µCT analysis was performed using a µCT50 (SCANCO, Brüttisellen, Switzerland). Radii were scanned at 70 kVp energy, 200 μA source current, 1000 projections per rotation, 800 ms integration time, and 17.2 µm voxel size. In order to spatially evaluate bone growth throughout the defect, bone area was calculated for each axial section (17.2 µm) totaling 20 mm. The area of interest (AOI) included the proximal onset of the defect and extended the length of the defect. It is not possible to distinguish AG from old or new bone utilizing µCT; thus, the ulna was included in analysis due to bone formation observed within the interosseous syndesmosis interfacing the ulna in some of the samples. The bone area was plotted as a function of defect length where 0 mm and 20 mm represented the proximal and distal ends of the defect, respectively. The bone volume (BV) and total volume (TV) within the AOI were measured to calculate the bone volume fraction (BV/TV). Additionally, trabecular thickening (Tb. Th.), trabecular spacing (Tb. Sp.), and trabecular number (Tb. N.) were evaluated.

### 2.6. Histological Evaluation

Non-decalcified histology was utilized to evaluate cellular infiltration, new bone formation, and residual polymer (*n* = 4 per treatment group) [38,42]. After formalin fixation, radii were dehydrated and embedded in poly (methyl methacrylate). Serial coronal sections were cut from the center of each defect with an Exakt band saw. Sections were polished and stained with Sanderson’s Rapid Bone Stain to assess osteogenesis and remodeling. Safranin O staining was also performed to assess endochondral ossification. An unstained section was utilized to analyze fluorochrome binding. High-magnification histological images were obtained via bright-field and fluorescent microscopy (Olympus BX41, Tokyo, Japan).

For quantitative histomorphometry, images were taken at 4× via-bright field and fluorescent microscopy (Biotek Cytation). The AOI was defined as a 20 × 5 mm rectangular region that encompassed the entirety of the graft and defect. The ulna was excluded from the AOI. The same AOI was used for both Sanderson’s Rapid stained and fluorescent sections. Quantification of new bone, infiltrating cells and tissue, and residual polymer was performed using Metamorph (Version 7.0.1). Bone was thresholded either as red (Sanderson’s rapid) or green/orange (fluorochromes). Residual material was thresholded as black stain, and infiltrating cells were thresholded as blue/teal. The thresholded area was reported as an area percentage of the total AOI.

### 2.7. Statistical Analysis

Data were analyzed utilizing GraphPad Prism (Version 8.4.1) and reported as mean ± standard deviation. Treatment group outcomes at 12 weeks were evaluated using an ANOVA with a Tukey’s multiple comparison test. Treatment group outcomes compared at 4 and 8 weeks were evaluated using a two-way ANOVA with Tukey’s multiple comparison test. Statistical significance was set at *p* < 0.05.

## 3. Results

### 3.1. Surgical Outcomes

**Figure 2:**
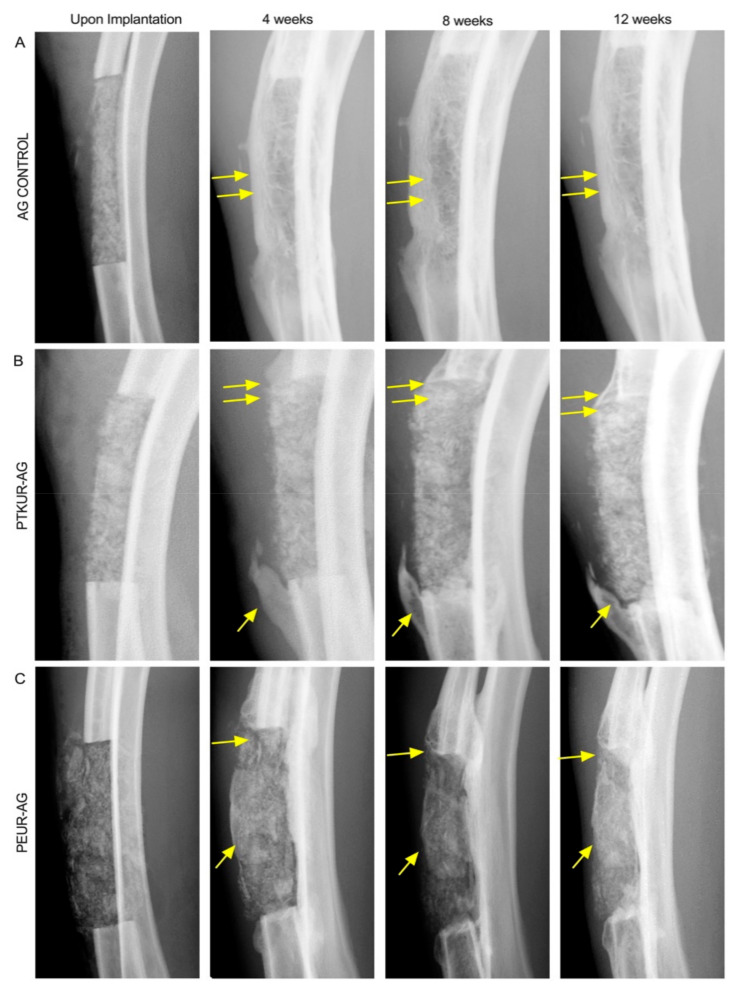
Representative X-ray images of (**A**) AG control, (**B**) PTKUR-AG, and (**C**) PEUR-AG acquired immediately after the surgical procedures and after 4, 8, and 12 weeks of healing. Areas of bone remodeling and formation are noted by yellow arrows.

The surgical procedures and subsequent healing were uneventful. No fractures of the radii occurred. As shown in Figure 2, X-rays displayed healing progression from 0 to 12 weeks in all of the groups. The AG control presented challenges in implantation and shape maintenance during the surgical procedures due to the lack of a settable polymeric extender. However, AG control remained in place throughout the study and displayed at least partial bridging of the defect along the radial side of the defect within three of the four samples (Figure 2A). Both AG extenders were coherent throughout surgical placement and remained stable throughout the entirety of the study (Figure 2B,C). The AG extenders displayed new bone growth at the host bone/graft interfaces, and graft remodeling was observed in both AG extenders, specifically near the proximal and distal ends of the defect where new bone and decreasing residual graft were observed. Bridging of the defect was not observed in any of the AG extender samples. Both AG extenders exhibited increasing opacity within the grafts over the 12-week time course, and no qualitative differences in new bone formation within the graft were observed between PTKUR- and PEUR-AG groups.

### 3.2. In Vivo Bone Analysis

**Figure 3:**
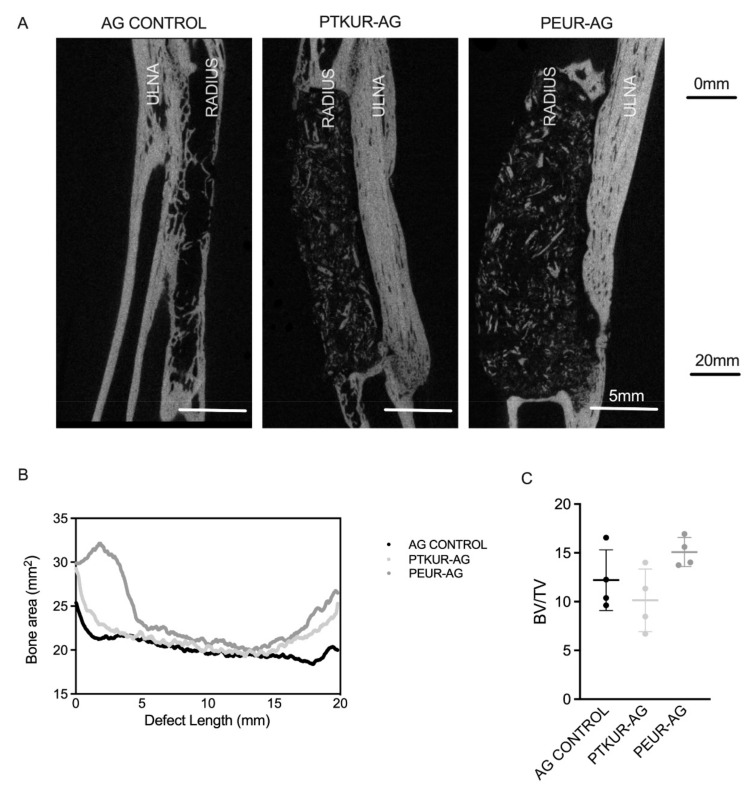
µCT analysis of bone remodeling. Representative µCT images of (**A**) AG control, PTKUR-AG, and PEUR-AG 12 weeks post-operatively. (**B**) Total bone area at 12 weeks measured as a function of defect length by µCT from the proximal to distal interfaces of the defect. Corresponding dotted lines representative standard deviation. (**C**) Bone volume/total volume (BV/TV) at 12 weeks for each treatment group.

Representative µCT images revealed no significant difference in total bone (including new bone, residual AG, and host bone) between groups at 12 weeks (Figure 3A). New bone formation was observed in the interosseous membrane in the space between the radius and ulna. Bone area and volume were quantified by µCT analysis (Figure 3B,C). All groups displayed similar trends of increased bone area at the proximal and distal ends of the defect with a gradual decrease in bone area as the center of the defect was approached (Figure 3B). BV/TV in PEUR-AG extenders trended higher compared with PTKUR-AG (*p* = 0.070) and AG control (*p* = 0.337), but the differences were not significant (Figure 3C). Additional bone morphometric parameters including trabecular thickness (Tb.Th.), trabecular separation (Tb.Sp.), and trabecular number (Tb.N.) did not show significant differences between groups (Supplementary Figure S2).

**Figure 4:**
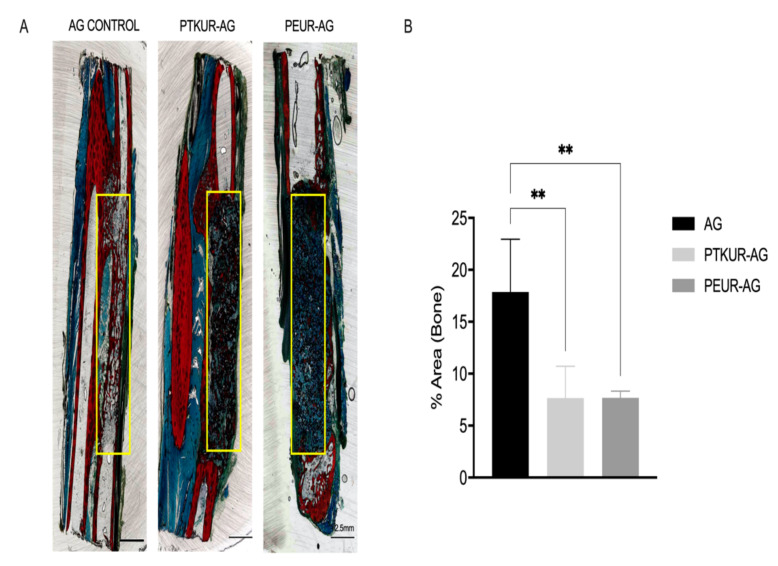
New bone formation in AG extenders. (**A**) Representative images of Sanderson’s Rapid stained AG control, PTKUR-AG, and PEUR-AG histological sections. The AOI (20 mm × 5 mm) used for analysis is indicated by the yellow box. (**B**) Histomorphometric analysis of area percentage of new bone (red) at 12 weeks within the defect. Statistical significance determined using one-way ANOVA, ** *p* < 0.01.

New bone formation measured via histological analysis was observed within all groups (Figure 4A and Figure S1). Quantitative histomorphometric analysis at 12 weeks showed no significant difference in new bone formation between PTKUR- and PEUR-AG extenders (Figure 4B). While µCT analysis showed no significant difference in BV/TV between the AG control and extender groups (Figure 3C), histomorphometric analysis showed significantly higher new bone formation compared with both AG extenders (Figure 4B). This discrepancy can be explained in part by the different regions of interest used for µCT (entire defect including the ulna) and histomorphometry (center of the defect excluding the ulna). Although the AG control displayed greater new bone formation, healing within the samples appeared to be inconsistent (Figure S1).

**Figure 5:**
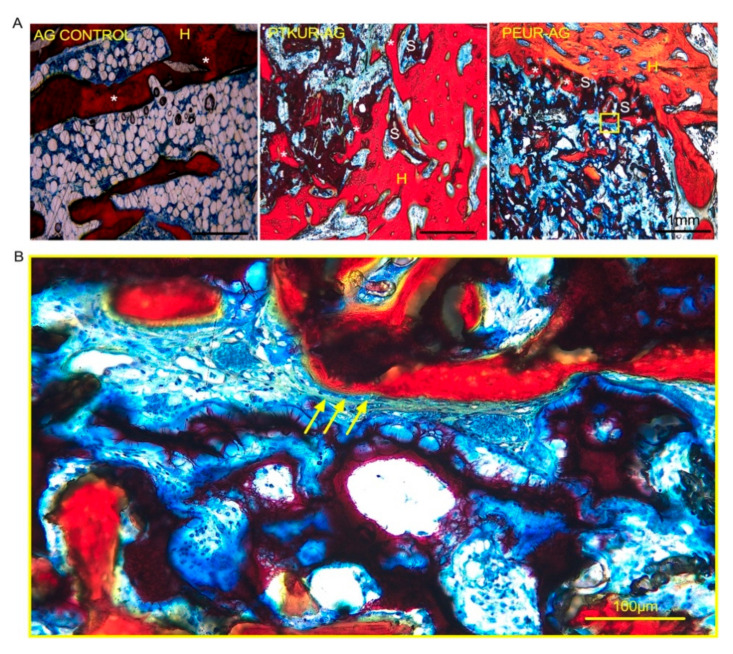
Osseointegration of AG extenders. (**A**) Histological images demonstrate osseointegration of the AG extenders at the host bone/material interface. H represents new bone, S represents scaffold, and * represents new bone growth. (Scale bar, 1 mm) (**B**) New bone growth occurring within the graft. Yellow arrows point to osteoblasts. (Scale bar, 100 µm).

Representative histological sections show the ingrowth of new bone at the graft interface indicating osseointegration in all groups (Figure 5A). While some specimens in the AG group showed increased adipogenesis in the marrow cavity compared with the extender groups (Figure 5A), images of histological sections from all AG specimens show variable adipogenesis (Supplementary Figure S1). Osteoblasts were observed around the perimeter of bone ingrowth, suggesting active ongoing remodeling (Figure 5B).

**Figure 6:**
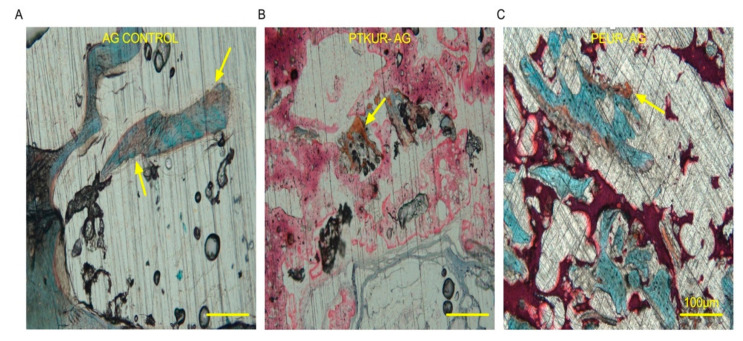
Endochondral ossification within AG extenders. Histological images demonstrate endochondral ossification with (**A**) AG control, (**B**) PTKUR-AG extenders, and (**C**) PEUR-AG extenders at 12 weeks. (Scale bar, 100 µm).

Additionally, Safranin O staining revealed faint orange staining (cartilage) indicating that previous endochondral ossification had occurred within the AG control group (Figure 6A), while ongoing endochondral ossification was observed at 12 weeks in both AG extenders (Figure 6B,C).

### 3.3. PTKUR and PEUR Graft Remodeling

**Figure 7:**
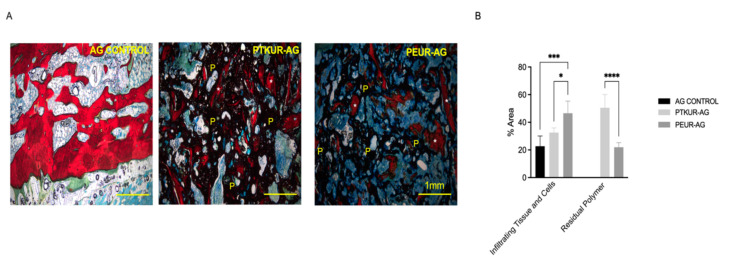
AG extender remodeling. (**A**) Representative images of residual polymer in AG control, PTKUR-AG, and PEUR-AG extenders. P denotes residual polymer and * denotes implanted AG. (Scale bar, 1 mm) (**B**) Histomorphometric analysis of area percentage of infiltrating cells and tissue and residual polymer within the defect after 12 weeks post implantation. Statistical significance determined using Two-way ANOVA, * *p* < 0.05, *** *p* < 0.001, **** *p* < 0.0001.

Histological analysis revealed residual polymer (black) in AG extenders (Figure 7A and Figure S1). High-magnification images demonstrated that PTKUR-AG underwent slower resorption as evidenced by the higher amount of dense residual polymer in the PTKUR-AG sections compared with the extensive resorption evident in the PEUR-AG sections (Figure 7A). These findings were confirmed via histomorphometric analysis (AOI represented in Figure 4A) in which PTKUR-AG exhibited significantly more residual polymer compared with the PEUR-AG group (Figure 7B). All groups supported cellular and tissue infiltration (teal/blue), but significantly greater cellular and tissue infiltration was observed in the PEUR-AG group compared with PTKUR-AG and AG control (Figure 7B).

**Figure 8:**
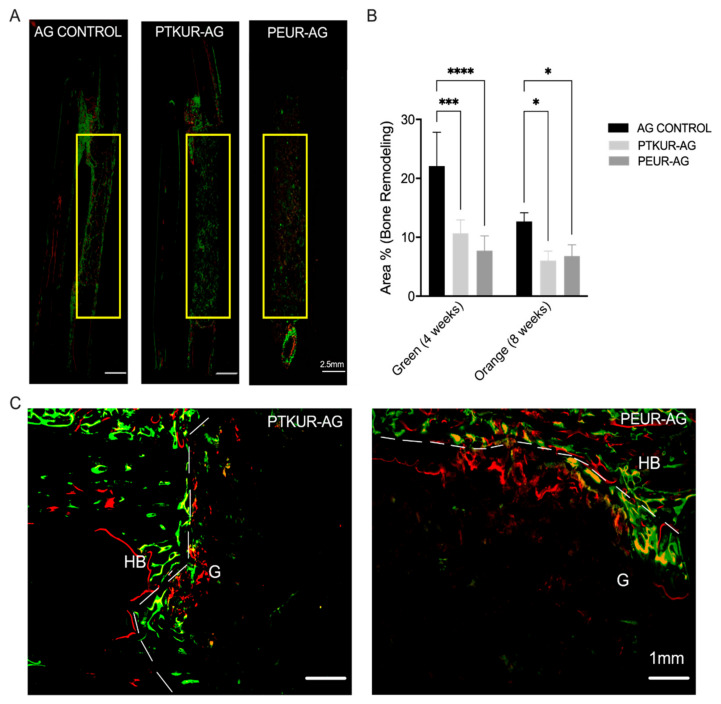
Dynamic histomorphometric analysis at 4 and 8 weeks. (**A**) Representative fluorescent images of AG, PTKUR-AG, and PEUR-AG groups. The AOI is indicated by the yellow box. (**B**) Histomorphometric analysis of area percentage of active bone remodeling at 4 (green) and 8 (orange/red) weeks within the defect. (**C**) Representative images of bone remodeling at the host bone–graft interface, demonstrating osseointegration in PTKUR-AG extenders and PEUR-AG extenders. HB indicates host bone and G indicates grafts. Statistical significance determined using two-way ANOVA, * *p* < 0.05, *** *p* < 0.005 **** *p* < 0.001.

Bone remodeling throughout the healing process was observed in all groups, especially at the proximal and distal host bone/graft interfaces (Figure S1). Remodeling was observed within the PTKUR- and PEUR-AG grafts around the periphery of implanted AG at 4 and 8 weeks, indicating mineralization nucleating from implanted AG particles within the extenders (Figure 8A). PTKUR- and PEUR-AG extenders exhibited increased bone remodeling at 4 weeks (green) compared with 8 weeks (orange/red); however, these differences were not significant (Figure 8B). Additionally, increased bone remodeling was observed at the graft/host bone interface, indicating the osseointegration of both AG extenders (Figure 8C). The AG control demonstrated significantly greater bone remodeling compared with the AG extenders at both 4 and 8 weeks (Figure 8B); however, inconsistent healing was observed as only two of the four controls exhibited complete bridging along the lateral edge of the defect (Figure S1).

## 4. Discussion

In this work, we implanted PTKUR-AG and PEUR-AG extenders in a 20 mm critical sized rabbit radial defect to evaluate the effects of polymer composition on both bone formation and graft remodeling in vivo. Both PTKUR- and PEUR-AG extenders supported new bone formation and utilized less AG than the AG control. Furthermore, the polymeric component of the AG extenders degraded and simultaneously maintained AG within the defect for 12 weeks. PEUR-AG extenders degraded more rapidly compared with PTKUR-AG extenders. However, new bone formation in both AG extenders was delayed compared with the AG control.

To understand the effect of polymer composition on bone formation and graft remodeling, AG extenders were implanted in a 20 mm critical-size radial defect in rabbits [44,45]. This model was selected as no external fixation was required [2]. No graft failure was observed in any of the groups throughout the 12 weeks, suggesting that AG extenders exhibited sufficient compression-resistant properties. Previous studies in the spine and mandible demonstrated that an elastic modulus >1 MPa provided compression-resistant properties [46,47]. We previously reported PTKUR-AG and PEUR-allograft moduli of 6.08 MPa and 4.38–9.47 MPa, respectively [39,48].

Previous studies performed in the rabbit radius have reported bone growth from the proximal end of the defect, the distal end of the defect, and the interosseus membrane [44,48,49,50,51]. Similarly, we observed bone formation in these directions. Due to the inability of µCT to distinguish between new bone, residual AG, host bone, and ossification extending from the ulna within the interosseous membrane to the radius, the ulna was included in µCT analysis. Interestingly, BV/TV trended higher in PEUR-AG compared with PTKUR-AG. These differences were not significant, but they were likely due in part to increased degradation of the PEUR, allowing for increased bone formation throughout the defect and within the interosseous membrane. Furthermore, µCT bone area quantification indicated increased bone at the proximal and distal end of the defect, indicating bone formation at the interfaces. Consistent with previous studies utilizing AG [52,53,54], new bone formation via creeping substitution at the host bone/graft interface was observed.

Histomorphometric analysis was performed to evaluate new bone formation specifically in the 5 mm × 20 mm defect space; thus, bone present in the interosseus membrane was excluded from analysis. Transverse sections were obtained from the center of the defect to evaluate bone formation at its most stringent point. Ultimately, no significant difference in bone between PTKUR- and PEUR-AG extender was observed via histomorphometry. The AG control demonstrated significantly increased new bone within the defect compared with AG extenders at 12 weeks via histomorphometric analysis, but new bone formation appeared to be variable throughout the defect. These differences were not observed in overall BV/TV between groups, suggesting that PTKUR- and PEUR-AG promoted bone formation, particularly in the interosseus membrane surrounding the defect while AG control promoted greater bone formation within the defect site itself.

In agreement with an earlier PTKUR-AG study in a biologically stringent intertransverse process defect [39], residual polymer was observed in PTKUR-AG extenders at 12 weeks. PEUR degradation occurred more rapidly than PTKUR degradation, as evidenced by significantly less residual polymer within the defect at 12 weeks. PTKUR degrades in response to specific cell types including osteoclasts, macrophages, and other ROS-secreting cells [38], while PEUR degrades via autocatalytic hydrolytic degradation [55]. Furthermore, AG control exhibited the least amount of cellular infiltration at 12 weeks, suggesting that cells recruited to the AG were osteoprogenitor cells that underwent direct differentiation. PTKUR-AG extenders exhibited less cellular infiltration than PEUR-AG extenders, demonstrating that cells were able to more readily infiltrate the graft as the polymer degraded. Early vascularization of cancellous AG begins at 2 days and is followed by the recruitment of MSCs in response to the osteoinductive signals of AG within the first weeks after implantation [56]. In contrast, AG particles were encapsulated in residual polymer in the AG extenders (Figure 5A), which delayed the rate of new bone formation. These findings are further confirmed by Safranin O staining in which faint positive Safranin O staining for cartilage was demonstrated within the AG control group, suggesting that new bone formation occurred via endochondral ossification and was near completion by 12 weeks. However, more intense positive Safranin O staining was observed in the AG extenders, suggesting that ongoing endochondral ossification was still occurring at 12 weeks. Thus, AG particles encapsulated in residual polymer retained their osteoinductivity beyond the first few weeks after implantation. Although the AG extenders showed delayed endochondral ossification suggesting a longer total healing time, the slower healing coupled with the observation that AG remains stable throughout the study suggests that PEUR and PTKUR extenders can reduce the risk for rapid resorption.

Dynamic bone histomorphometry is a widely utilized method for evaluating bone remodeling [57,58]. As mentioned above, ossification within the interosseus membrane was excluded from dynamic histomorphometric analysis. In agreement with our static histomorphometric findings, bone remodeling at 4 and 8 weeks was greater in AG controls compared with PTKUR- and PEUR-AG extenders within the defect. It is likely that the lack of polymer in AG controls allowed for more extensive cellular infiltration and new bone formation. Despite a smaller 10 mm defect size, a previous study utilizing highly porous cell-seeded hydroxyapatite scaffolds did not observe fluorochrome binding within the scaffold until six weeks post implantation [51]. Herein, fluorescent staining beginning at 4 weeks was apparent within the grafts in the AG extender groups, suggesting that embedded AG maintained bioactivity. Additionally, abundant osseointegration at the host bone/graft interface in both AG extenders was observed, further confirming bioactivity. AG is resorbed by osteoclasts and new bone is deposited by osteoblasts in a process known as creeping substitution [56].

The complete degradation of synthetic polymers requires from 4 to 24 months in vivo [59], which can delay the creeping substitution of AG particles encapsulated in polymer. However, while the encapsulation of AG particles within residual polymer delays new bone formation, it also protects AG from rapid resorption that can result in unpredictable healing [10]. While osteobiologics such as recombinant human bone morphogenetic protein-2 (rhBMP-2) have been shown to promote predictable bone healing at 12 weeks in the rabbit radius defect model [60], rhBMP-2 is approved by the FDA for only a limited number of indications, including lumbar fusion, ridge augmentation, and fresh fractures of the tibial shaft. Furthermore, rhBMP-2 delivered on a collagen sponge has weak mechanical properties, similar to AG. Thus, PEUR- and PTKUR-AG extenders may be most beneficial in clinical scenarios where long-term mechanical stability is required, such as posterolateral spine fusion and fractures of the mid-diaphysis. This study is limited by a single intermediate time point for endpoint outcomes (12 weeks), at which time bone healing and complete resorption of residual polymer and AG were not observed. Future studies should focus on optimizing the rate of polymer degradation to increase the rate of new bone formation while protecting the AG from excessive resorption.

## 5. Conclusions

In this work, PTKUR- and PEUR-AG extenders were compared with an AG control in a rabbit radius model of bone regeneration. PTKUR- and PEUR-AG extenders both maintained AG in the defect throughout the study and demonstrated bone formation along the host bone/graft interface comparable to AG control. Polymer resorption and subsequent cellular infiltration were observed within the defect space in both AG extenders but did not have an effect on overall bone formation. These results suggest that early polymer degradation and cellular infiltration are necessary for harnessing and maximizing the osteoinductive capabilities of AG.

## Data Availability

The data presented in this study are available on request from the corresponding author. The data are not publicly available due to privacy restrictions.

## Supplementary Materials

The following are available online at https://www.mdpi.com/article/10.3390/ma14143960/s1, Figure S1: Fluorescent and Sanderson’s Rapid stained histological sections. Figure S2: Bone morphometric parameters.
