# Supplementary material for: Settable Polymeric Autograft Extenders in a Rabbit Radius Model of Bone Formation
Source: Materials (Basel). 2021 Jul 15;14(14):3960. doi: 10.3390/ma14143960 (PMC8305944; doi:10.3390/ma14143960)
Supplement: Supplementary file 1 [file materials-14-03960-s001.zip › materials-1262641-supplementary.pdf]

materials

Article

Settable Polymeric Autograft Extenders in a Rabbit Radius Model of Bone Formation

Lauren A. Boller [1], Madison A.P. McGough [1], Stefanie M. Shiels [2], Craig L. Duvall [1], Joseph C. Wenke [2] and Scott A. Guelcher [1,3,4,*]

[1] Department of Biomedical Engineering, Vanderbilt University, 2201 West End Ave, Nashville, TN 37235, USA; lauen.a.boller@vanderbilt.edu (L.A.B.); madison.a.mcgough@gmail.com (M.A.P.M.); craig.duvall@vanderbilt.edu (C.L.D.)
[2] U.S. Army Institute of Surgical Research, 3698 Chambers Rd, San Antonio, TX 78234, USA; stefanie.m.shiels.ctr@mail.mil (S.M.S.); joseph.c.wenke.civ@mail.mil (J.C.W.)
[3] Department of Chemical and Biomolecular Engineering, Vanderbilt University, 2201 West End Ave, Nashville, TN 37235, USA
[4] Vanderbilt Center for Bone Biology, Vanderbilt University Medical Center, 1211 Medical Center Dr., Nashville, TN 37212, USA
* Correspondence: scott.guelcher@vanderbilt.edu

materials

Supplemental Figures

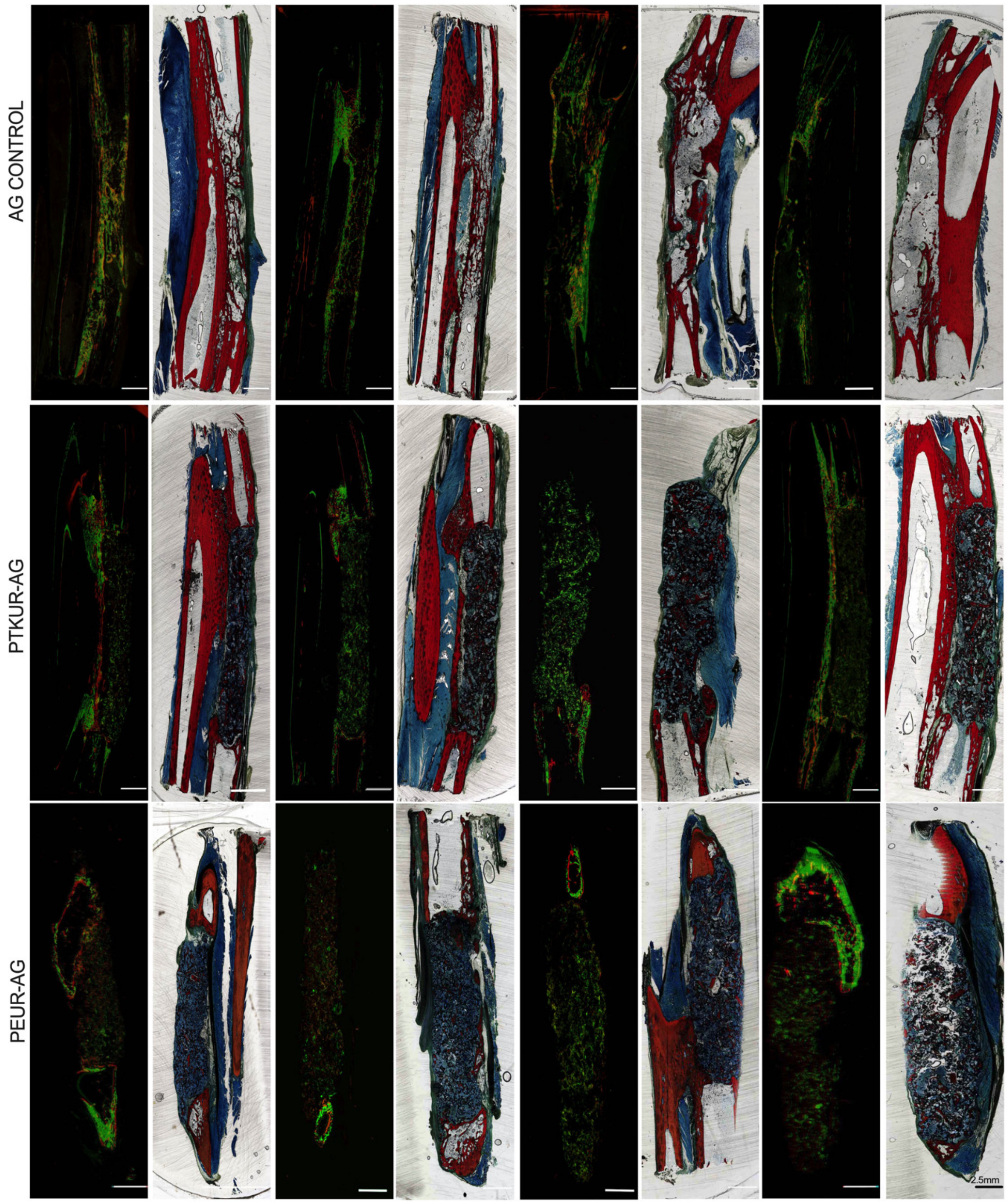

Figure S1. Fluorescent and Sanderson's Rapid stained histological sections.

materials

MDPI

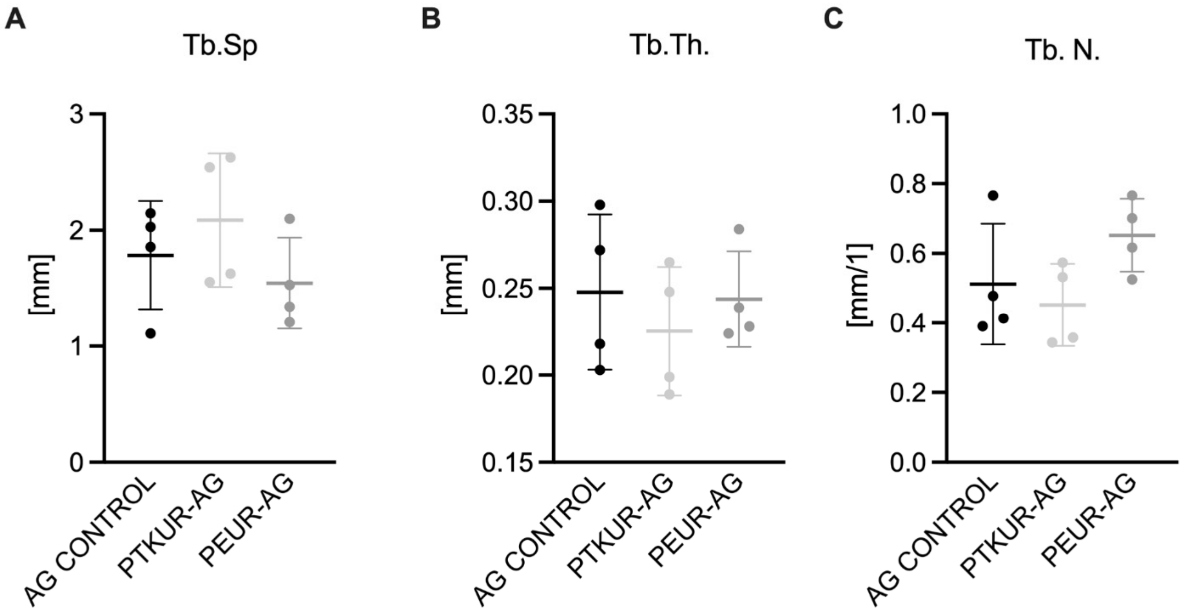

Figure S2. Bone Morphometric parameters A) Trabecular Spacing, B) Trabecular Thickening, and C) Trabecular Number.
